# Public involvement in the dissemination of the North West Coast Household Health Survey: Experiences and lessons of co‐producing research together

**DOI:** 10.1111/hex.12940

**Published:** 2019-07-29

**Authors:** Clarissa Giebel, Shaima Hassan, Jason C McIntyre, Rhiannon Corcoran, Ben Barr, Mark Gabbay, Jennifer Downing, Terence Comerford, Ana Alfirevic

**Affiliations:** ^1^ Institute of Psychology, Health and Society University of Liverpool Liverpool UK; ^2^ Collaboration for Leadership in Applied Health Research and Care North West Coast Liverpool UK; ^3^ School of Natural Sciences and Psychology Liverpool John Moore's University Liverpool UK; ^4^ Institute of Translational Medicine University of Liverpool Liverpool UK

**Keywords:** co‐production, dissemination, health, health inequalities, public involvement, survey

## Abstract

**Background:**

Engaging with the public is a key element of health research; however, little work has examined experiences of public involvement in research dissemination. The aim of this paper was to assess the extent of public involvement, experiences of public advisers and resulting changes in the dissemination of the North West Coast Household Health Survey (HHS).

**Methods:**

Three writing groups allowed public advisers to contribute to the dissemination of the HHS. A public workshop was set up to aid the co‐production of the research evidence and discuss the experiences of public advisers involved with the survey in March 2018. A focus group with public advisers was conducted in August 2018 to understand their experiences of involvement. Data were analysed using thematic analysis and coded by two researchers. Writing groups are still on‐going.

**Results:**

Fourteen public advisers contributed via three face‐to‐face writing groups, by actively interpreting findings and helping in the write‐up of research articles and by presenting talks at the public workshop. At the workshop, seven public advisors contributed to setting priorities for data analysis from the HHS. Five public advisers took part in the focus group, which highlighted that whilst public advisers were generally satisfied with their involvement, they would like to be involved in more activities.

**Conclusions:**

Members of the public shaped the dissemination of evidence and provided guidance for future steps. Public advisers were mostly positive about their involvement in the dissemination of the HHS, but highlighted the need for more transparency and support from researchers.

## 1 INTRODUCTION

Patient and public involvement (PPI) is becoming increasingly important in the design and conduct of health research, and is strongly recommended to be an integral part of the research process.^1^ PPI enables members of the public with lived experiences of a health‐care condition such as dementia or service history such as mental health treatments to actively shape research. PPI can take many shapes and forms, from identifying research priorities^2^, ^3^ to developing questionnaires and interpreting and disseminating findings.^4^, ^5^ Past research suggests that members of the public have reported to feel that their contributions have had a positive impact on shaping health research.^6^ The benefits of engaging the public are not restricted to the actual research project, however; as being involved as a member of the public can also increase self‐confidence and allow people to improve their personal skill sets.^7^

The North West Coast region contains some of the most disadvantaged neighbourhoods in England.^8^ It has been found that being from a low socio‐economic background, which can include unemployment, low income, low levels of education and belonging to a minority ethnic group, is associated with receiving different levels of access to and utilization of health‐care services.^9^, ^10^ This can be due to distances to services, but also due to reduced levels of health literacy, and thus knowing when and where to access the right services and which types of services are available.^11^, ^12^

The North West Coast Household Health Survey (HHS) is implemented and supported by the National Institute for Health Research (NIHR) Collaboration for Leadership in Applied Health Research and Care North West Coast (CLAHRC NWC). The survey examined 20 disadvantaged neighbourhoods and eight less disadvantaged neighbourhoods within the region and is one of the first surveys to combine socio‐economic data with data on mental and physical health as well as health‐care service utilization in a large‐scale public health survey. By comparing disadvantaged with less disadvantaged neighbourhoods, the objective of the survey was to assess health inequalities and identify ways to improve suitable health‐care access. Comprising two waves of data collection, the second wave was completed by the end of 2018, with the first wave having collected data on 4319 residents. Members of the public have been involved in all stages of the concept, design, conduct and analysis process of the Household Health Survey (HHS).

For PPI to be effective, INVOLVE^1^ recommends adhering to six core values: Public advisers and their opinions need to be *respected* (a) and *supported* (b), with researchers being *transparent* (c) about the intended and possible involvement within the research. Researchers should *respond to* (d) the issues raised by the public and indicate where changes have been made to the research. The involvement overall should be as *diverse* (e) as possible to ensure that a wide range of opinions and experiences are captured to help shape the research. Lastly, researchers are *accountable* (f) and should feedback on the involvement to those who have contributed their time as public advisers to the research. These guidelines ensure that the input provided by the public closes in a full circle, so that public advisers also receive some benefits and feedback from the researchers to better understand how their input has impacted on the research.

This paper had two aims: (a) to assess the extent of public involvement and (b) to explore the experiences of public advisers in the dissemination of the HHS. This was framed around the NIHR INVOLVE guidelines on public involvement^1^ and by assessing the public advisers’ opinions on their level of involvement via a focus group. To date, there is limited evidence on the level of involvement of members of the public specifically in the dissemination of research, as reports mostly focus on a project as a whole.^13^ Involving the public in the dissemination of the results of this survey is vital to reach residents from both disadvantaged and relatively advantaged backgrounds, and thus help share knowledge and discuss how health inequalities could be reduced, which is a very important area of public involvement.^14^

## 2 METHODS

For the purpose of disseminating the findings from the NWC Household Health Survey, a group was set up to have oversight of all activities—the Healthcare Utilisation Group. This group fostered the co‐production of research together with members of the public and aimed to adhere to the six principles of good public involvement as outlined by INVOLVE.^1^ These included respect, support, transparency, responsiveness, diversity and accountability. We sought to maximize the level of public involvement in the writing groups and a half‐day public workshop, and have explored their experiences of their in a focus group setting.

Writing groups have taken place since 2017 and are still on‐going. The workshop took place in March 2018, and the focus group took place in August 2018.

### 2.1 Recruitment

Members of the public were recruited via different pathways. Some were recruited via different NHS Trusts and services, and others were recruited from an existing contact list of interested members of the public from the CLAHRC NWC, who had previously not participated in any CLAHRC activities but had merely expressed their interest in becoming a public adviser. In addition, some public advisers were recruited via word of mouth.

In particular, public advisers were allocated into the three writing groups to provide input into specific topics. For example, members were recruited for papers focusing on old age who were a member of the public who either accessed older adult clinical services themselves or worked with older adults. Similarly, people with personal or indirect experience of mental health problems were recruited this way and then allocated into the specific writing group in which this analysis and paper were being held.

### 2.2 Focus group

To gather the experiences of members of the public and their levels of satisfaction of involvement in the Healthcare Utilisation Group, a 1‐hour focus group was held at the University of Liverpool. People were recruited via email from the list of public advisers involved in the Healthcare Utilisation Group, and five public advisers attended the focus group (three female and two male). Participants were asked in particular about their activities in the Healthcare Utilisation Group and about their positive and negative experiences so far. In addition, participants were asked about whether they had been given sufficient opportunities to be involved and asked for recommendations regarding how to increase their involvement and how to guide new public advisers into their role. The focus group was audio‐recorded and subsequently transcribed, and data were analysed using thematic analysis^15^ by two members of the research team (CG and SH), who had previously been trained in conducting qualitative analysis. Both researchers coded the data separately and subsequently generated final codes via discussion and agreed on main overarching themes.

## 3 FINDINGS

### 3.1 The extent of public involvement in the dissemination of the HHS

Members of the public were involved both in writing groups and in a public dissemination event as part of the dissemination of the HHS, to help shape the research reporting of findings from the NWC HHS.

### 3.2 Shaping research in writing groups

In order to improve the structure of the dissemination, people were allocated into three writing groups, led by two research team members (CG and JD). Writing Group 1 focused on findings relating specifically to *socio‐economic factors*, such as socio‐economic predictors of Accident and Emergency attendance and social inequalities in serious infectious diseases. Writing Group 2 focused on findings relating to *mental health*, such as the links between primary care use and mental health and differences in treatment according to levels of disadvantage. Writing Group 3 focused on findings related to *physical health*, including co‐morbidities and complex needs.

A total of 51 people are members of the Healthcare Utilisation Group, and its three writing groups. Of these, 15 are partners from NHS Trusts and local authorities, 22 are academics, and 14 are public advisers. Of those 14 public advisers, eight are female and two are from a minority ethnic background. Members of the public represented various backgrounds with a variety of experiences in the topics being investigated by the HHS, including members of Black, Asian and ethnic minority groups, and people who had extensive experience as patients of health services. Prior to joining the writing groups, members of the public were made aware of their expected roles and responsibilities, which included attending research meetings and contributing to various aspects of the dissemination process, such as interpreting findings and reading through drafts of manuscripts. To ensure a *transparent* and *responsive* approach, the expectations of the public advisers were clearly stated on role descriptions and agreed within each writing group. The role description of the public advisers is attached in Appendix 1. Each writing group meets every few months to update on the progress of individual analyses and interpret the findings, lasting approximately 90 minutes. To date, a total of 19 writing group meetings have taken place.

Within the writing groups, public advisers engaged in a wide range of activities related to analysis and dissemination. During research meetings, public advisers actively shared their opinions on the results from Wave 1 of the HHS and helped to interpret those results from their personal perspective. In addition, public advisers provided feedback on drafts of academic papers and therefore co‐produced research as co‐authors on publications.^11^ To ensure that findings from the HHS were not solely restricted to the academic sector, public advisers were trained and *supported* to write up the findings in a format suitable for lay audiences, which have been published online and circulated to other members of the public. This training involved guiding them through the writing process, either by sitting down with the public adviser and drawing out the main points of the research to be noted down, or by providing guided feedback on their lay summaries. If public advisers were unable to attend writing group meetings, they are being sent the documents via email. Minutes of the meetings were circulated to group members to record and demonstrate how the research team has integrated the opinions and thoughts of the public advisers.

### 3.3 Public workshop

To involve members of the public to a greater extent in the dissemination of the findings, we organized a co‐production workshop comprising public advisers, partners from local authorities and NHS Trusts, as well as academics. In total, 21 participants attended the workshop, of which seven were public advisers. The aim of the workshop was to disseminate some of the current findings, jointly interpret some of these findings, set priorities and identify new analyses. Some public advisers were from the neighbourhoods sampled in the research, which ensured that the research team *accounted* to the communities and neighbourhoods affected by the research. The objective of the workshop was twofold: the first was to disseminate the first wave of findings from the survey to a wider audience; the second was to ask attendees for their thoughts on strategies and topics to explore with the data and therefore raise the level of co‐production of the dissemination of the findings.

To ensure that public advisers not only contributed to the dissemination event, but were also *supported* to form an active part of the event itself, one public adviser gave a talk about his experiences of being involved in the HHS. For this purpose, the public adviser was guided on how to give a presentation and ensure he felt confident in doing so.

At the workshop, people were provided with topics from the three writing groups (socio‐economic factors, mental health and physical health) and asked to prioritize topics for dissemination, such as multiple high risk factors of A&E attendance and policy implications. In particular, attendees discussed and identified priorities first at their group tables, which was followed by a general discussion, in which all priorities were highlighted and discussed to establish the most important ones. Attendees were also asked to discuss future priorities of the collected data, which may have received little attention to date. Across all groups, attendees identified a list of topics that could either be explored with the existing data from Wave 1 of the survey or potentially should be addressed in future waves. Some of the priority areas identified by the groups for analyses included exploring the risk factors of GP attendance in disadvantaged neighbourhoods; detailed investigation of long‐term conditions; and focusing on the policy implications for mental health. Specifically, mental health as a topic area was considered one of the most important to address, and whilst each group table was asked to focus on different topics, mental health as a priority area resonated from all group discussions.

Considering the emphasis on mental health analyses and their implications for practice and policy, attendees unanimously recommended to hold a series of similar workshops in the future but with more specific foci. Specifically, it was proposed that one workshop should solely focus on analyses and findings surrounding mental health. A separate workshop should focus on analyses and findings surrounding Accident and Emergency attendance and the role of socio‐economic influences. Additional workshops were suggested to focus on physical health, as well as health inequalities as a general concept. By implementing these workshops, the public will not only to a greater extent be involved in co‐producing research, but the workshops can also enable wider dissemination of findings to relevant stakeholders, such as local authorities and policymakers.

Implementation in general was considered a high, but often neglected, priority in research. With some of the analyses currently on‐going, and some of the first findings emerging, attendees of the workshop expressed a great desire to see changes in real life as a result of the survey findings. As part of this, it was important to prioritize interventions that had the potential to reduce health inequalities. Attendees pointed out that these changes may differ between different communities and neighbourhoods, so that implementation will need to be guided further by close co‐production with local public advisers and relevant organizations. Whilst the CLAHRC NWC ensures that all findings are accessible and disseminated to the wider public, for example in the form of lay handouts and social media stories, attendees wanted the findings to have an impact on policy and to be implemented in their local neighbourhoods. In sum, there was a desire among attendees to find a better balance between understanding phenomena and focussing on implementation in communities.

To ensure *transparency*, attendees were informed of the outcomes of the workshop via email and were informed how their ideas and thoughts are being addressed in the next step of the survey. By actively making changes to the dissemination of the HHS, their thoughts were *respected*.

### 3.4 The experiences of public advisers in the dissemination of the HHS

At the focus group, public advisers shared their experiences of being involved in the dissemination of the HHS findings. All attendees were part of a writing group, and all had attended the workshop. Overall, the focus group feedback suggested public advisors felt positive about their involvement. Three main themes emerged from the data analysis: developing new skills, need for support and guidance, and transparency in research.

#### 3.4.1 Developing new skills

Participants felt that being part of this group provided them with opportunities to develop new skills and strengthen their confidence. As part of these new skills, being involved also *supported* people with potential language barriers to become more confident in using the English language, which showcases the aim of involving people from *diverse* backgrounds, and enabling people from different minority groups of becoming involved:

I feel hesitation to speak because I think my English is not that good […] I want to be involved, because it gives me positivity in so many different ways, like I meet different people. It gives me confidence. I can't speak much but it gives me confidence gives me a positive influence. (P2)

I'm really enjoying it and its bringing like you were saying different skills. (P5)

#### 3.4.2 Need for support and guidance

Whilst the qualitative analysis suggested the overall experience of advisers was positive, public advisers expressed the need for better support at the beginning of their journey as a public adviser. In particular, some public advisers felt they should have received more guidance of what they would be expected to do as part of the group. On a logistical level, they would have preferred better directions to meetings and more accessible buildings. Indeed, several meetings were held at university campuses, which can be difficult to navigate. A buddy system for new public advisers was suggested, whereby a new public adviser will be connected with an existing public adviser, who can help them get to meetings, go into meetings jointly and help them with any peer support they may need:

When some newcomer is joining our team we need to tell them what they're going to do because for me, when I joined it I don't know what to do. If there's a buddy system so we need to tell them ok this, this thing and you need to […] you know tell them what are you going to do here. (P4)

they may need support, they may need some guidance or maybe some training to find tune the skills that they have and that's from the person that's very quiet and is not involved in anything right through to the people that are involved in lots of groups. (P1)

#### 3.4.3 Transparency in research

Along with better guidance from the beginning, public advisers would wish to see more *transparency* in the academics’ approach to research and co‐production and better communication of the objectives of the group and individual dissemination activities. Some public advisers felt unsure after some meetings why they had attended the meetings, and what benefits they have provided to the meeting:

I think it would be nice if when we do these things that not only will (we say) ‘this is the study’ but it would be […] ‘this is the objective’, this is what we're trying to achieve overall and where this study will go towards working towards that objective do you understand. (P1)

I hope I had some useful input into that verbally but I must admit at times I do feel a little bit that I'm lost. (P5)

## 4 DISCUSSION

The aim of this paper was to evaluate both the extent of public involvement and experiences of public advisers in the dissemination of the HHS. Findings showed that public involvement was overall considered positive and beneficial to the overall dissemination, highlighting how public advisers felt supported and respected, and how researchers ensured accountability, transparency, responsiveness and diversity, where possible. We also learned several lessons from this work, which should be addressed to further improve the experiences of public advisers.

In light of the NIHR INVOLVE guidelines on public involvement, the dissemination of the HHS has and currently is incorporating many of the elements of good public involvement in its work. This includes being *respectful* with one another and enabling people from *diverse* backgrounds to become involved. A previous evaluation of public involvement in a large 5‐year long research project has highlighted how important it is to have more social diversity within the public advisers.^16^ Whilst we only had two public advisers from minority ethnic backgrounds, these public advisers have contributed significantly to shaping the dissemination to date. In future, we need to ensure to recruit more members of the public from diverse backgrounds. Indeed, including people with different experiences and different backgrounds can help us better understand how to prioritize, conduct and disseminate research findings for different groups in everyday life.

Whilst training is provided to public advisers and they are thus *supported* in their involvement, such as when presenting at the workshop, public advisers felt that they should receive more support at the beginning of their involvement. As a result of this feedback, we are planning on setting up a buddy system, as suggested in the focus group, for future public advisers. This is also corroborated by a recently published model on public involvement in dementia research,^17^ which clearly highlights various ways in which people with dementia specifically (or generally public advisers) should be involved with research. In particular, the authors, which included three independent groups of people living with dementia, and thus members of the public, highlighted the need for on‐going training and support underpinning all aspects of a public adviser’s contribution to a research project, ranging from designing and collecting data to understanding and disseminating the findings. In addition, public advisers from the HHS expressed a need for academics to be more *transparent* with the objectives of meetings and individual activities to avoid any confusion about their benefits or contributions. This has been also picked up in a recent study on public involvement across England, suggesting that researchers need to provide more feedback to public advisers.^18^ Therefore, we will be clearer about the objectives of meetings and activities from the beginning prior to the activity, so that public advisers can decide whether this is relevant to them.

Lastly, by addressing the expressed wishes for further support as well as the outcomes of the workshop and focusing on implementing the findings in the local communities, we aim to be *responsive* by taking action on public adviser recommendations, as well as take *accountability* and share our findings with those neighbourhoods that were involved in this research. This is an important step to avoid a tokenistic approach to public involvement, and instead work together with public advisers as equals as part of the research team, something that has often been criticized in previous public involvement activities across health research.^19^ In particular, the workshop has provided several steps to be undertaken simultaneously. First, attendees recommended to hold a series of topic‐specific workshops, such as on mental health, health‐care utilization or hospital admissions. By holding further workshops and making these more accessible to those communities that were involved in the survey, we can increase the *accountability* of this survey. Second, the present research suggests we need to prioritize specific analyses as recommended by workshop attendees, particularly those focusing on mental health, which generated the most public interest. In line with these recommendations, we will be investigating new topics such as self‐harm and will be implementing findings in practice to help reduce health inequalities. These changes to our dissemination and overall translation of evidence clearly showcase the positive impact that public involvement has had and is still having in the dissemination of the HHS, supporting previous evidence by public advisers on the impact and value of their public involvement in health research.^6^ Moreover, this highlights the value of involving the public in order to reduce inequalities in health^14^ and also their value in the implementation of the findings due to their knowledge of the local communities, which is corroborated by previous evidence.^20^, ^21^ As a result, one priority will be to accumulate all findings from a mental health perspective and draft a policy paper with guidelines for policy and potential pathways for implementation.

This study has some limitations. Whilst all public advisers who are part of the writing groups and who have attended the workshop were invited to attend the focus group, not all were able to attend. Considering their different levels of involvements in the writing groups and for different analyses, not all public advisers attended the writing groups to which they were allocated regularly. Some only contributed in more specific analyses during team meetings due to time constraints. Therefore, findings on the experiences of public involvement in the dissemination of the HHS are limited to those public advisers who were frequent attendees of the writing groups and possibly more motivated. However, findings were not all positive and highlighted areas for improvement, indicating that focus group attendees highlighted a range of experiences.

## 5 CONCLUSIONS

This present evaluation has successfully addressed the aim of evaluating the extent of public involvement and the experience of public advisers in the dissemination of the findings of the HHS. In particular, this evaluation has shown that members of the public are overall positive about their involvement in the dissemination of the HHS, but have also highlighted areas for improvement in line with NIHR INVOLVE^1^ guidelines, including for the research team to be more *transparent* in their expectations and in providing more *support* to new public advisers. Co‐producing research with members of the public has provided valuable insights into what research should be prioritized, how to maximize public involvement and satisfaction with their involvement and how we can improve co‐production practices in relation to research dissemination, thereby addressing a previously identified gap on the impact of public involvement in different research stages.^4^ Findings from this study suggest that public involvement in the dissemination of research should encompass a variety of activities to enable different members of the public to become involved, such as through writing groups and co‐production workshops. It is hoped this work will improve research stemming from the HHS going forward and provide guidance to those co‐producing research with members of the public.

## CONFLICT OF INTEREST

There are no conflicts of interest.
