# Supplementary material for: Public involvement in the dissemination of the North West Coast Household Health Survey: Experiences and lessons of co‐producing research together
Source: Health Expect. 2019 Jul 29;22(4):643–9. doi: 10.1111/hex.12940 (PMC6737839; doi:10.1111/hex.12940)
Supplement: Supplementary file 1 [file HEX-22-643-s001.docx]

**Collaboration for Leadership in Applied Health Research and Care North West Coast NIHR CLAHRC NWC**

**Theme: Cross-Theme**

**Project: Integrated Longitudinal Research Resource (ILRR)**

**Role Title: Dissemination Public Advisor**

**Role Description:**

**Background –** The NIHR CLAHRC NWC is a collaboration of thirty seven partners including twenty NHS organisations, nine Local Authorities, three Universities (University of Central Lancashire, Lancaster University and University of Liverpool) and members of the public across the North West coast – from Cumbria to Cheshire. The overall aim of the CLAHRC is to help to reduce the inequalities in health and life expectancy found across the NW coast area (and improve people’s health).

To support this work we are bringing together a wide range of data and statistics (for example, from surveys, the national census and hospital data) into a single resource called the Integrated Longitudinal Research Resource (ILRR). We aim to use this information to track changes in the social, economic and environmental factors that impact on people’s health, and to investigate what health interventions have worked and what could be done differently.

We aim to use this collection of information to produce academic publications, share information with Local Authority Partners (who make decisions that affect neighbourhoods in the North West), and to inform the public of these key research findings also.

**The Role of Dissemination Public Advisors** - is to work as part of a team with health practitioners, local authority representatives, and core CLARHC staff and researchers to produce pieces of work using information from the ILRR. These pieces of work may include academic publications, CLAHRC BITES, reports, press releases, and presentations. You will be asked to share your views as a member of the public, you are not expected to have specialist knowledge about health or research. You will have the opportunity to be involved in the research in a variety of ways. You need to agree the level of involvement you wish to have with your engager and the support required for this (e.g. training, mentor etc).

**Role and Duties**

Dissemination Public Advisors will be able to:

a) Attend dissemination meetings (organized on an ad hoc basis by project leads);

b) Provide comments and advice on the readability of reports and to offer advice on who might benefit from receiving the information.

c) With support from staff, assist with writing publications, presenting findings, and analysing data.

d) Be a resident of the CLAHRC NWC region.

All public advisor contributions to a project or piece of work will be acknowledged in any publications, reports or presentations produced. This can be done by including you as an author or within the acknowledgement section depending upon your contribution and your own preferences.
